# Have a good day! An experience-sampling study of daily meaningful and pleasant activities

**DOI:** 10.3389/fpsyg.2022.977687

**Published:** 2022-11-16

**Authors:** Christoph Kreiss, Tatjana Schnell

**Affiliations:** ^1^Existential Psychology Lab, Institute of Psychology, University of Innsbruck, Innsbruck, Austria; ^2^Psychology of Religion and Existential Psychology, MF Norwegian School of Theology, Religion and Society, Oslo, Norway

**Keywords:** meaning in life, meaningfulness, pleasure, everyday life, activity, hierarchical meaning model, experience sampling, arts

## Abstract

We organize our daily lives with a relatively high degree of freedom. Some things must be done; others are optional. Some we find meaningful, some pleasant, some both, and some neither. The present study looks at such evaluations of daily activities and how they relate to perceived meaning in life. Sixty-two students from an Austrian university first completed the *meaningfulness* scale from the Sources of Meaning and Meaning in Life Questionnaire (SoMe). They then participated in a 1-week experience-sampling assessment, wherein they completed a short questionnaire at five random time-points per day. They indicated their current activity and then reported, on a 6-point Likert scale, how pleasant and meaningful they perceived it to be. Activities could thus be categorized as meaningful, pleasant, both, or neither. Results reflected that activities grouped under *culture/music, communication, intimacy,* and *sports* are experienced as both highly meaningful and pleasant. A two-level hierarchical linear regression suggested that people with high trait meaningfulness experience their daily activities as more meaningful than people with lower trait meaningfulness if they also enjoy what they are doing. People with low trait meaningfulness, however, tended to experience their daily activities as rather meaningless, even if they enjoyed them very much. Thus, when looking for advice on how to have a good day, clarifying one’s meaning in life seems to represent the best starting point.

## Introduction

In Western Countries, daily life is largely de-traditionalized; social norms leave large parts of private life untouched. Thus, when determining what makes life meaningful, the classical authorities (such as the state or the church) have lost their power over the individual. When it comes to managing our daily lives, we are mostly left to our own devices. Moreover, in heavily individualistic societies, lifestyle choices are multi-optional: Members can—and must—choose from an overwhelming number of possibilities like lifestyles, jobs, relation types, etc. (Gross, 1994). Yet individuals are not offered answers on how to achieve a good life in the eudaimonic sense (i.e., a meaningful life), and so they instead strive for maximizing pleasure (Christensen, 2017; Schnell, 2022). Exclusive pursuit of pleasure can take on an addictive nature (Bechara, 2005), and bring about unhappiness (Mauss et al., 2011, 2012; Ford et al., 2014, 2015), whereas a meaningful life is associated with many positive outcomes, of which happiness appears to be a by-product (Schueller and Seligman, 2010; Vötter and Schnell, 2019a). People who have a stronger sense of meaning in life tend to be, not only happier, but also more socially involved and engaged (Stavrova and Luhmann, 2016). Furthermore, they are more hopeful and optimistic (Damásio et al., 2013); feel more self-determined (Kashdan and Breen, 2007); are more self-forgiving (Vötter and Schnell, 2019b); have higher self-efficacy, resilience, and self-regulation; and are better able to motivate, activate, calm themselves, direct their attention, and cope with failure (Hanfstingl, 2013; Sørensen et al., 2019; Schnell and Krampe, 2020, 2022). The correlates of trait meaningfulness are thus well-researched. However, less is known about how people experience meaning during individual activities or over the course of a day (King and Hicks, 2021).

### Why is it worth investigating meaning in everyday life?

Several studies indicate that meaning is not a stable construct, but changes from moment to moment and from day to day (King et al., 2006; Choi et al., 2017; Martela et al., 2018). Newman et al. (2021) observed that it made a difference whether people were asked to rate the meaningfulness of their lives as a whole or across just one day. In particular, the degree of reported meaning differed significantly between these conditions. This may have been due to memory effects, which according to Newman et al. have a major influence on one’s evaluation of life as meaningful. It also seems that peak experiences—such as graduation, marriage, or childbirth—are considered when assessing the meaningfulness of one’s life. For these reasons, global judgments of meaning significantly overestimate meaning judgments collected *in situ* (Newman et al., 2021).

### What makes a good day?

As to the question of what makes a good day, one possible answer could lie in our activities: how we experience them and why we engage in them. In an experience-sampling study, Huta and Ryan (2010) revealed that people mostly reported either hedonic or eudaimonic motives for their activities, but not both at the same time: those two motive types were negatively correlated on the within-person level. When the scores were aggregated to obtain between-person estimates, however, hedonic and eudaimonic motives were correlated positively. This suggests that people who are highly motivated by eudaimonic pursuits also tend to report hedonic interest. In the long run, a combination of both motive types predicted higher levels on several measures of wellbeing. This finding was confirmed by Delle Fave et al. (2011); they demonstrated that the people who experienced the highest levels of wellbeing, physical and mental health, and life satisfaction were those who sought pleasure and meaning simultaneously. Christensen (2017) offers a neuroscientific explanation for the benefits of engaging in both meaningful and pleasant activities: Two systems associated with pleasure and meaning can be distinguished in the brain. The first is the “A-system” consisting of amygdala, posterior ventromedial prefrontal cortex (VMPFC), and striatum (including nucleus accumbens). It functions as the brain’s reward system and is responsible for maximizing feelings of immediate gratification. Secondly, there is the “I-system” consisting of the insula, anterior VMPFC, hippocampus, dorsolateral prefrontal cortex, and anterior cingulate cortex; it is responsible for maximizing deferred reward and personal growth. Exclusively pleasant activities (e.g., eating fast food or watching a trivial film) activate the A-system, whereas activities that are typically associated with meaningfulness (e.g., studying or working) activate the I-system (Bechara, 2005; Rolls, 2015). Christensen (2017) argues that ideally, these two systems should be activated simultaneously. The A-system tempts us to do the same activities repetitively, but this can lead to dependence on maladaptive reward-seeking strategies, such as smoking cigarettes, eating fast food, or gambling. However, if we exclusively seek out activities which trigger the I-system, it can cause a loss of desire for these activities and hinder their execution, even though the relative benefits for mental and physical health are clear. For this reason, she posits that we should connect the A- and I-systems by engaging in activities which are not only conducive to wellbeing, but which also create a desire to repeat them. She concludes her argument with the so-called “arts hypothesis.” It suggests that only the arts are perceived as meaningful and pleasant at the same time, since they activate both the A- and I-systems. However, this overemphasis of the arts is viewed critically by others, since no receptor or area in the brain has yet been discovered to respond solely to the arts (Skov and Nadal, 2018). Offering a similar two-path approach, Choi et al. (2017) illustrated that various activities could be differentially—but not exclusively—associated with meaning and *happiness*. By their account, people can perceive different undertakings as having one of four possible permutations of these two elements: happy-meaningful, happy-meaningless, unhappy-meaningful, or unhappy-meaningless.

In consideration of these dual-aspect (eudaimonic-hedonic) paradigms, the present study focused on the experience of activities as pleasant and/or meaningful. We first tested the aforementioned arts hypothesis, which suggests that only artistic activities are experienced as both pleasant and meaningful at the same time (hypothesis 1). In doing so, we also examined whether experiences of meaningfulness and pleasantness can occur simultaneously in activities, as indicated by Choi et al.’s (2017) finding concerning happiness and meaning (hypothesis 2).

### The hierarchical meaning model

The Hierarchical Meaning Model (HMM) by Schnell (2009, 2014, 2021) is a pyramid model in which the upper levels influence the lower levels, and vice versa. The top of the model represents meaning in life, operationalized by the two dimensions of meaningfulness and crisis of meaning. This is followed by the sources of meaning, goals, actions, and perception. In the present study, we focus on the influence of meaningfulness on actions and perception. According to the HMM, people with a high degree of trait meaningfulness should experience their daily activities as more meaningful than people with low trait meaningfulness (hypothesis 3). As shown by King et al. (2006) and Martela et al. (2018), positive feelings also contribute to the experience of meaning. We therefore also investigated the extent to which perceived state pleasantness—as an indicator of positive affect—predicts the perception of state meaningfulness and vice versa. For both analyses, the moderating effect of trait meaningfulness was tested: Given varying degrees of trait meaningfulness, how does the evaluation of the meaningfulness of activities differ, depending on their pleasantness? And how does the evaluation of the pleasantness of activities differ, depending on their meaningfulness (exploratory analysis 1)? Finally, we explored whether people with high (contrasted with low) trait meaningfulness differed in the type of activities performed (exploratory analysis 2). These results may be particularly informative regarding the practical question of how to live a eudaimonic daily life.

## Materials and methods

### Samples

In a pretest, *N* = 30 students (70% female, mean age 22 years, SD = 1) participated in a 1-week diary study. At the end of each day, they had the task of listing, in a digital document, the activities they had engaged in that day. The totality of all mentioned activities was then summarized and categorized by the last author and a colleague. A catalog of 13 activity types ensued: study, work, household, eating, resting, watching TV, sports, reading, communication, culture/music, intimacy, transit-time, and other. In the following step, this catalog was used in the Experience-Sampling Method (ESM) study presented here. A sample of *N* = 62 students (80% female, mean age 23 years, SD = 4) were involved in this study. No sample size planning was done prior to the survey; instead, the aim was to recruit as many participants as possible.

### Procedure and measures

The participants first completed the meaningfulness scale from the Sources of Meaning and Meaning in Life Questionnaire (SoMe, Schnell and Becker, 2007), using a paper-pencil version. They then participated in a 1-week ESM study, wherein we used a palmtop for collecting the data. This device beeped five times a day at random times to prompt participants to respond to a short survey. At these time-points, they were then asked to select the type of activity in which they were currently engaged from the pretest-derived list, and then subjects were to rate, on a Likert scale from 0 (*strongly disagree*) to 5 (*strongly agree*), how meaningful and how pleasant they perceived the activity to be. Pleasantness was measured by a single item (“It is pleasant.“), whereas the meaningfulness of the activity was assessed by a five-item scale, adapted from the SoMe (Schnell and Becker, 2007). The items on this adapted version were: “I experience it as meaningful.,” “It fits my life task.,” “It fulfils me.,” “It makes me feel like I am part of something bigger.,” and “It has a deeper meaning..”

## Results

The gathered data were analyzed using *R-Studio* version 4.2.0. and *IBM SPSS Statistics* version 26. The following software packages in *R-Studio* were used: *ggplot2* (Hadley, 2016), *lme4* (Bates et al. 2018), *interactions* (Long and Long, 2019), *nlme* (Pinheiro et al. 2017), *psych* (Revelle, 2018), *dplyr* (Wickham et al. 2019b), and *tidyverse* (Wickham et al. 2019a).

### Descriptive statistics

The ESM survey had a response rate of 85% for the entire week and across all subjects. The number of observations, means, standard deviations, skewness, kurtosis, range, and Cronbach’s alpha is presented in Table 1. Skewness and kurtosis values for all variables indicated a near-normal data distribution (<|2|, George and Mallery, 2020). Inter-variable correlations ranged from *r* = 0.10 to *r* = 0.66 (see Table 2). Applying a cutoff score of 3 for perceived state pleasantness/meaningfulness (Schnell, 2010), participants experienced 52% of their activities as pleasant and 40% of their activities as meaningful.

**Table 1:** Descriptive statistics for trait meaningfulness, state meaningfulness, and state pleasantness.

Scale	*n*	*M*	SD	Skewness	Kurtosis	Cronbach’s alpha
Trait meaningfulness	62	3.30	0.98	−0.41	−0.60	0.82
State meaningfulness	1,863	2.55	1.20	−0.03	−0.70	0.87
State pleasantness	1,863	3.40	1.34	−0.64	−0.23	−^a^

*n* = datapoints, *M* = mean, SD = Standard deviation. Range for all scales: 0–5.

a One item only.

**Table 2:** Intercorrelations of trait meaningfulness, state meaningfulness, and state pleasantness.

Variables	Trait meaningfulness	State meaningfulness
State meaningfulness	0.37**	
State pleasantness	0.10**	0.44**

^**^p < 0.01.

Table 3 shows the proportion of variance at the between-person (level 2) and within-person (level 1) levels for state meaningfulness and state pleasantness. The data are organized in a hierarchical dataset, where situations (level 1) are nested within persons (level 2). Most of the variance in state pleasantness (86%) and, to a lesser degree, in state meaningfulness (62%) can be attributed to situational variation (within-person variance). Comparatively, more than a third (38%) of the variance in state meaningfulness could be explained by individual differences (between-person variance).

**Table 3:** Proportions of variance at the between-person (level 2) and within-person (level 1) levels for state meaningfulness and state pleasantness.

	State meaningfulness	State pleasantness
Person (level 2)	38%	14%
Situation (level 1)	62%	86%
Total	100%	100%

Situation (level 1) nested in persons (level 2).

### Hypothesis 1

Hypothesis 1—the arts hypothesis—postulated that only cultural activities are experienced as both highly meaningful and highly pleasant. We tested whether the participants assessed cultural activities as more meaningful and pleasant than all other activities by conducting two two-level hierarchical linear regression models (HLM), in which level 1 represented situations and level 2, persons. The activity “culture/music” served as a reference group for all other activities. An activity was considered different from culture/music if the unstandardized regression coefficients were significantly different. The final model contained a random intercept, which allowed activities to vary from person to person, and since there were no continuous predictors in the model, the slope could not be random and was thus fixed. Activity types constituted the independent variable, while state meaningfulness and state pleasantness served as the dependent variables. Although there was evidence of heteroscedasticity, HLM is considered a relatively robust procedure (Darandari, 2004). All other relevant assumptions of this statistical model were tested prior to analysis and were deemed to have been satisfied, as supported by the values in Table 1. The results, as shown in Table 4, indicate that cultural activities were indeed experienced as meaningful and pleasant simultaneously. However, this combination was not unique to cultural pursuits, as it was also observed for three other types of activities, namely: communication, sports, and intimacy.

**Table 4:** Descriptive statistics of state meaningfulness and state pleasantness across daily activities and results of the two two-level hierarchical linear regressions with the outcome variables state meaningfulness and state pleasantness predicted by daily activities, all at level 1, nested in persons at level 2.

				State meaningfulness				State pleasantness			
	*N*	*n*		*M*	SD	*b* (SE)	95% CI	*M*	SD	*b* (SE)	95% CI
Culture/music	34	60	(3.27%)	3.24	0.94			4.13	0.92		
											
Communication	52	212	(11.55%)	3.01	1.23	−0.11 (0.12)	[−0.35, 0.13]	4.33	0.82	−0.09 (0.16)	[−0.40, 0.22]
Doing housework	45	101	(5.50%)	1.83	0.96	−1.41 (0.14)***	[−1.67, −1.15]	2.12	1.27	−2.10 (0.17)***	[−2.44, −1.76]
Eating	59	184	(10.02%)	2.63	1.03	−0.63 (0.12)***	[−0.87, −0.39]	4.11	0.92	−0.16 (0.16)	[−0.47, 0.15]
Intimacy	17	30	(1.63%)	3.21	1.27	−0.03 (0.19)	[−0.39, 0.34]	4.17	1.15	−0.19 (0.24)	[−0.66, 0.27]
Other	43	124	(6.75%)	2.00	1.26	−1.07 (0.13)***	[−1.32, −0.81]	3.26	1.46	−0.92 (0.17)***	[−1.25, −0.59]
Sports	22	35	(1.90%)	3.40	1.27	0.09 (0.18)	[−0.26, 0.44]	4.26	0.85	−0.09 (0.23)	[−0.54, 0.36]
Reading	25	46	(2.51%)	2.92	1.06	−0.43 (0.16)**	[−0.74, −0.11]	4.02	0.93	−0.36 (0.21)	[−0.76, 0.05]
Relaxing	59	218	(11.87%)	2.25	1.10	−0.87 (0.12)***	[−1.11, −0.64]	3.95	1.25	−0.20 (0.16)	[−0.51, 0.10]
Studying	61	458	(24.95%)	2.93	1.03	−0.16 (0.11)	[−0.38, 0.06]	2.62	1.18	−1.61 (0.15)***	[−1.90, −1.33]
Transit-time	50	127	(6.92%)	2.18	1.16	−1.08 (0.13)***	[−1.33, −0.83]	2.90	1.22	−1.39 (0.17)***	[−1.71, −1.06]
Watching TV	52	162	(8.82%)	1.61	1.00	−1.50 (0.13)***	[−1.75, −1.26]	3.84	1.09	−0.42 (0.16)**	[−0.73, −0.10]
Working	21	79	(4.30%)	2.60	1.47	−0.26 (0.15)	[−0.55,0.03]	2.89	1.26	−1.13 (0.19)***	[−1.50, −0.76]
Total	62	1,836	(100%)	2.55	1.20			3.40	1.34		

*N* = number of subjects who carried out the activity; *n* = number of occurrences of the activity; *M* = mean; SD = standard deviation; b = unstandardized regression coefficients for state meaningfulness and pleasantness at level 1; SE = Standard Error; 95% CI = 95% Confidence Intervals of regression coefficients for state meaningfulness and pleasantness. **p < 0.01, ***p < 0.001. −2*Log Likelihood (−2LL) of the final model of state meaningfulness = 4,619.84*** and −2LL of state pleasantness = 5,462.28***. Pairwise comparisons of −2LL by ANOVAS were used to determine model gains. Activities were effect coded. The activity “culture/music” was used as the baseline. The final model had a random intercept with activities as predictor. Activities (level 1) were nested in persons (level2). Intercept with 95% CI for state meaningfulness = 3.15 (0.14)*** [2.87, 3.43]; intercept with 95% CI for state pleasantness = 4.22 (0.15)*** [3.93, 4.52].

### Hypothesis 2

The next hypothesis aimed to check the assumption that activities can be experienced as both meaningful and pleasant, which was examined with the same analyses used to test Hypothesis 1. The data modeled and reported in Table 4 supports this assumption, and Table 5 shows which types of activities were considered meaningless-unpleasant, meaningful-unpleasant, meaningless-pleasant, and meaningful-pleasant.

**Table 5:** Four types of activities: pleasant-meaningful, unpleasant-meaningful, pleasant-meaningless, unpleasant-meaningless.

	Unpleasant activity	Pleasant activity
Meaningless activity	Doing housework, other, transit-time	Eating, relaxing, reading, watching TV
Meaningful activity	Studying, working	Communication, culture/music, intimacy, sports

### Hypothesis 3

Our third hypothesis intuited that people high in trait meaningfulness also tend to perceive their momentary engagements as more meaningful. To address the multi-level structure of this research question, we again utilized a two-level hierarchical linear regression. Level 1 represented situations and level 2, persons, with the situations level nested within persons. Trait meaningfulness served as a predictor for the outcome variable, state meaningfulness. Given that correlation analyses indicated a positive relationship between state meaningfulness and state pleasantness, the latter was included as a covariate. As suggested by Antonakis et al. (2021), we further controlled for person-average pleasantness. The final model (model 4) allowed the person level to have a random intercept of the outcome variable, state meaningfulness, and the regression slopes were allowed to vary freely from one participant to the next. The results from this analysis (as presented in Table 6) indicate that trait meaningfulness (*b* = 0.25, SE = 0.05, 95% CI [0.14, 0.35], *p* < 0.001); person-average pleasantness (*b* = 0.51, SE = 0.09, 95% CI [0.33, 0.70], *p* < 0.001); and state pleasantness (*b* = 0.30, SE = 0.03, 95% CI [0.24, 0.36], *p* < 0.001) all predicted the experience of situations as meaningful—independently of the activity types in which participants engaged.

**Table 6:** Two-level linear regression results; outcome variable: state meaningfulness.

	Model 1	Model 2	Model 3	Model 4^a^	Model 5	Model 6^b^
	*b* (SE)	*b* (SE)	*b* (SE)	*b* (SE)	*b* (SE)	*b* (SE)
*Level 2 (person)*						
Intercept	2.55 (0.10)***	1.07 (0.28)***	−1.12 (0.39)**	−1.01 (0.32)**	−0.78 (0.89)	−0.50 (0.89)
Trait meaningfulness (tm)		0.45 (0.08)***	0.34 (0.06)***	0.25 (0.05)***	0.17 (0.28)	0.10 (0.28)
Person-average pleasantness (pap)			0.75 (0.11)***	0.51 (0.09)***	0.44 (0.27)	0.52 (0.27)
*Interaction (level 2)*						
tm*pap					0.02 (0.08)	0.00 (0.08)
*Level 1 (situation)*						
State pleasantness (sp)				0.30 (0.03)***	0.30 (0.03)***	0.07 (0.11)
*Cross-level interaction*						
tm*sp						0.07 (0.03)*
*Model fit*						
−2LL	5,195.09	5,169.94***	5,136.24***	4,759.35***	4,759.27	4,754.2*

^*^p < 0.05, ^**^p < 0.01, ^***^p < 0.001; *b* = unstandardized regression coefficients, SE = standard error, −2LL = −2*Log Likelihood. Pairwise comparisons of −2LL by ANOVAS were used to determine model gains. Model 1 = Intercept-Only-Model with random intercept per person, Model 2 = Random Intercept with trait meaningfulness (level 2) as predictor with random intercept per person, Model 3 = Random Intercept with trait meaningfulness (level 2) and person-average pleasantness per person as predictor (level 2) with random intercept per person, Model 4 = Random-Slope Model with trait meaningfulness (level 2), person-average pleasantness (level 2) and state pleasantness (level 1) as predictors with random intercept per person and state pleasantness as random slope, Model 5 = Random-Slope Model with trait meaningfulness (level 2), person-average pleasantness (level 2), state pleasantness (level 1) and the interaction on level 2 between trait meaningfulness and person-average pleasantness as predictors with random intercept per person and state pleasantness as random slope, Model 6 = Random-Slope Model with trait meaningfulness (level 2), person-average pleasantness (level 2), state pleasantness (level 1) and the cross-level interaction between trait meaningfulness (level 2) and state pleasantness (level 1), interaction on level 2 between trait meaningfulness and person-average pleasantness as predictors with random intercept per person and state pleasantness as random slope.

a Final model, hypothesis 3.

b Final model, exploratory analysis 1.

### Exploratory analysis 1

We followed up the previous findings with an exploratory analysis, wherein we investigated whether state pleasantness was predictive of perceived state meaningfulness, when an individual’s degree of trait meaningfulness is considered as a qualification of the model from hypothesis 3. This analysis was designed to elucidate the relationship between the predictor variable (trait meaningfulness) and its covariates (person-average pleasantness and state pleasantness). The final model 6 (see also Table 6) was a cross-level interaction model with trait meaningfulness (level 2) as a moderator for the relation between state pleasantness (level 1) and state meaningfulness (level 1). According to this model, the tendency to experience situations as meaningful was only positively predicted by the interaction of trait meaningfulness and state pleasantness (*b* = 0.07, SE *=* 0.03, 95% CI [0.01, 0.13], *p* < 0.05) As this cross-level interaction remained significant after including the interaction between trait meaningfulness and person-average pleasantness on level 2, it can be deemed a true cross-level interaction (Antonakis et al., 2021).

**Figure 1:**
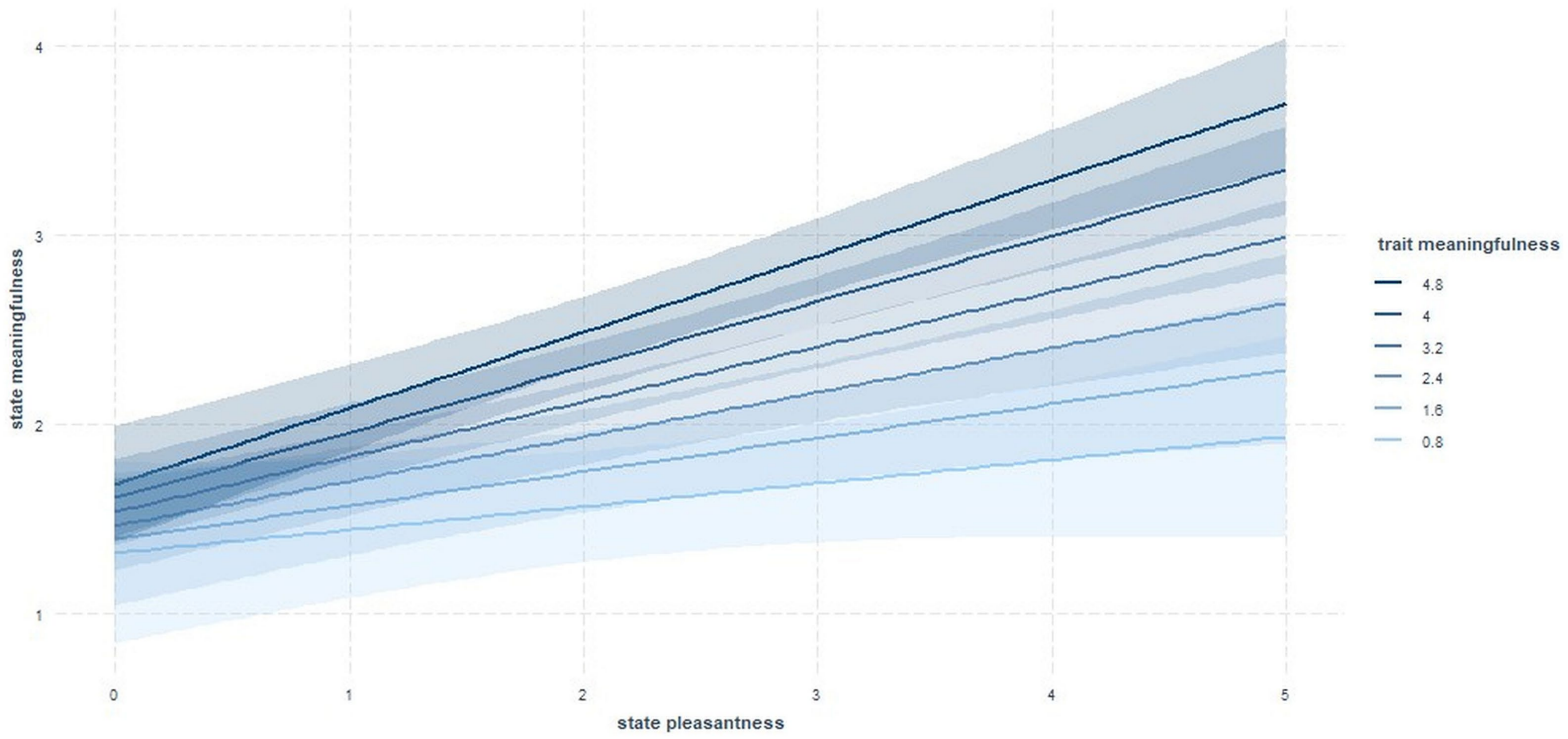
Interaction of trait meaningfulness and state pleasantness in the prediction of state meaningfulness.

**Figure 2:**
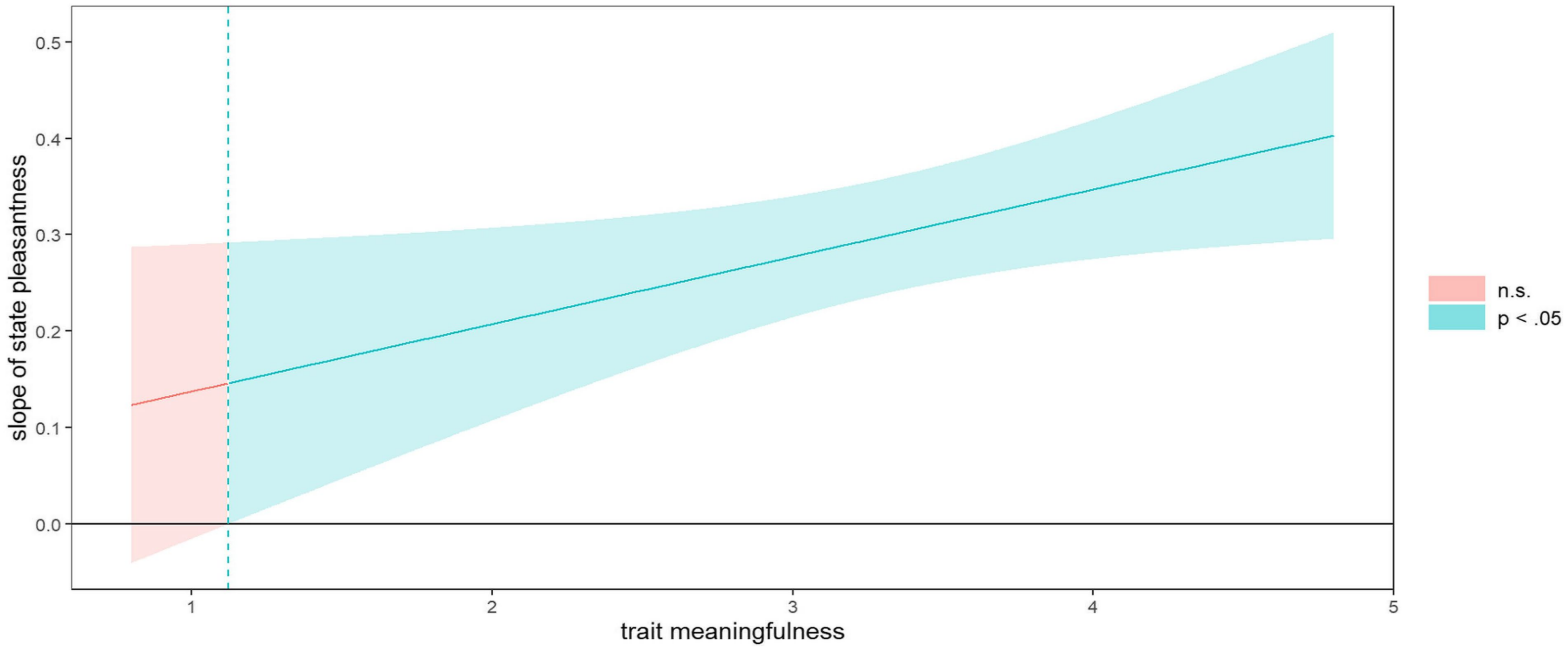
Johnson Neyman plot of interaction trait meaningfulness and state pleasantness.

Figure 1 illustrates the interaction between the independent variable (state pleasantness) and the moderating variable, trait meaningfulness. The Johnson Neyman plot (Figure 2) indicates significant results for all parts of this interaction, except for the lowest section: For moderator (trait meaningfulness) values below 1.17, state pleasantness did not significantly interact with trait meaningfulness. In other words, the more the activities were perceived as pleasant, the more they tended to also be experienced as meaningful. This correlation was clearly more pronounced among participants who tested high for trait meaningfulness. Among those with low trait meaningfulness, state meaningfulness did not covary with state pleasantness. With regard to absolute values, the data show that individuals with low trait meaningfulness (<3) viewed their present endeavors as little meaningful on the whole (average scores did not exceed 2.9, see Figure 1), even if they also rated those same actions as very pleasant.

In a similar vein, we examined whether state meaningfulness tended to predict reported state pleasantness, when we have accounted for the participant’s trait meaningfulness. The final model incorporated state meaningfulness as random slope and random intercepts per person; the predictors modeled were trait meaningfulness, person-average meaningfulness, and state meaningfulness. The analysis yielded no significant model gain by adding the interaction between state meaningfulness and trait meaningfulness. As Model 4 in Table 7 shows, state pleasantness was positively predicted by state meaningfulness (*b* = 0.49, SE = 0.04, 95% CI [0.41, 0.58], *p* < 0.001), but not by trait meaningfulness nor by person-average meaningfulness.

**Table 7:** Two-level linear regression results; outcome variable: state pleasantness.

	Model 1	Model 2	Model 3	Model 4
	*b* (SE)	*b* (SE)	*b* (SE)	*b* (SE)
*Level 2 (person)*				
Intercept	3.40 (0.07)***	2.94 (0.24)***	2.34 (0.20)***	2.15 (0.23)***
Trait meaningfulness		0.14 (0.07)	−0.11 (0.07)	−0.11 (0.06)
Person-average meaningfulness			0.56 (0.08)***	0.12 (0.08)
*Level 1 (situation)*				
State meaningfulness				0.49 (0.04)***
*Model fit*				
−2LL	6,109.94	6,106.06*	6,072.34***	5,738.30***

^*^p < 0.05, ^**^p < 0.01, ^***^p < 0.001; *b* = unstandardized regression coefficients, SE = standard error, −2LL = −2*Log Likelihood. Pairwise comparisons of −2LL by ANOVAS were used to determine model gains. Model 1 = Intercept-Only-Model with random intercepts per person, Model 2 = Random Intercept with trait meaningfulness (level 2) as predictor with random intercepts per person, Model 3 = Random Intercept with trait meaningfulness (level 2) and person-average meaningfulness (level 2) as predictor with random intercepts per person, Model 4 = Random-Slope Model with trait meaningfulness (level 2), person-average meaningfulness (level 2) and state meaningfulness (level 1) as predictors with random intercepts per person and state meaningfulness as random slope.

### Exploratory analysis 2

Finally, by means of a Chi-square test, we tested whether people with a high level of trait meaningfulness (scores ≥ 3; *cf.* Schnell, 2010) engaged in more pleasant-meaningful activities, i.e., those found in the evaluation of hypothesis 1 (e.g., *culture/music, communication, intimacy, sports*; see Table 5). The results indicated that this was not the case: Participants with high trait meaningfulness did not seem to differ significantly from participants with low trait meaningfulness in terms of the frequency of carrying out activities typically rated as pleasant-meaningful.

## Discussion

Based on the combination of a questionnaire survey and a 1-week experience-sampling study, our data provided several insights into how people with high trait meaningfulness spend and experience their days. Depending on the type, activities were experienced as meaningless-unpleasant, meaningful-unpleasant, meaningless-pleasant, or meaningful-pleasant. Our analysis results led to a rejection of Christensen’s (Christensen, 2017) hypothesis that only artistic activities are experienced as both pleasant and meaningful. Instead, the data implied that not just culture and music are experienced as simultaneously meaningful and pleasant, but also doing sports, sharing intimacy, and communicating. Similar results were reported by Choi et al. (2017) for combinations of happiness and meaningfulness. They found that various activities such as cooking, dating, eating, sports, playing an instrument, praying, reading, shopping, socializing, traveling, walking, communicating, and volunteering were associated with both meaning and happiness at once. To observe a common denominator, pleasant-meaningful activities all seem to be self-selected (leisure) activities. This ties in with findings from Martela et al. (2018), who suggested that the fulfillment of basic psychological needs (autonomy, competence, and relatedness) predicts daily meaningfulness.

In contrast to state meaningfulness (62%), state pleasantness was more affected by the current situation (86%). This suggests that state pleasantness is more volatile than state meaningfulness. This might be one of the reasons why the pursuit of happiness has been found to cause unhappiness (Mauss et al., 2011, 2012; Ford et al., 2014, 2015), as people who only chase quick happiness are much more dependent on factors that are external to themselves – and thus, beyond their control. Further research should clarify this volatility hypothesis.

A substantial overlap between situational pleasure and meaning was indicated by the positive correlation (r = 0.44) between state pleasantness and state meaningfulness. This result fits in with previous research on the close connection between positive feelings and meaning (King et al., 2006; Hicks et al., 2012; Tov and Lee, 2016; Chu et al., 2020; Miao and Gan, 2020).

Trait meaningfulness and state meaningfulness were moderately correlated (*r* = 0.37) with each other. This suggests that people with a higher sense of meaning in life also perceive their daily activities as more meaningful; but neither can evaluations of meaning in life be determined from momentary experiences, nor vice versa. A similar conclusion was already reached by Newman et al. (2021) with reference to global and repeated daily assessments of meaning. Moreover, in both Newman et al. (2021) and the present data, global judgments overestimated aggregated daily and situational states. When assessing global meaning in life, people appear to adopt a meta-perspective that allows them to weight and re-evaluate individual experiences (Schnell, 2021). In the assessment of everyday situations, i.e., immersed in the midst of life, this perspective seems to be often unavailable—which is also reflected in the fact that these situations are assessed as less meaningful overall.

Then, we confirmed through mixed-model analysis that one’s perceived meaning in life statistically predicts the way in which they experience everyday activities. Our reported evidence for hypothesis 3 provides further empirical support for the HMM by Schnell (2009, 2014, 2021), which states that a general sense of meaning increases the perception of daily actions as meaningful. The moderator analysis for the prediction of state meaningfulness revealed another interesting result: When the interaction of state pleasantness and trait meaningfulness was included, the respective unique contributions of the two variables were no longer significant. This leads us to qualify the results for hypothesis 3: people with high trait meaningfulness do not always perceive activities as more meaningful, but rather, only when they are experienced as at least somewhat pleasant. Unpleasant activities are, in principle, more likely to be regarded as meaningless, irrespective of a person’s general sense of meaning in life. The significant predictor person-average pleasantness suggests that, overall, people who generally enjoy their pursuits are prone to also find them more meaningful. Nevertheless, the interaction between person-average pleasantness and trait meaningfulness was not significant, which is an indicator that we found a true cross-level interaction between trait meaningfulness and state pleasantness. We interpret this finding to indicate that trait meaningfulness predicts daily actions (in the sense of the HMM) regardless of whether people generally enjoy their daily actions more or less.

Whereas Halusic and King (2013) concluded that pleasure is sufficient, but not necessary, for the experience of state meaningfulness, our data suggest that positive feelings might be predictive of perceiving activities as meaningful (at least among people with high trait meaningfulness), but they are not sufficient. People with low trait meaningfulness evaluated their daily activities as low in meaning, even if they experienced them as very pleasant.

Conversely, the pleasantness of an activity was predicted by one’s tendency to experience that same activity as meaningful, but not by trait meaningfulness, nor by their interaction. This suggests that meaningful activities are often experienced as pleasant as well. In these cases, we might apply Klinger’s (Klinger, 1998) explanation: positive feelings have a confirmatory utility for experiencing meaning. They provide positive feedback that something meaningful has been accomplished.

When we finally explored the question if people high in trait meaning engaged more often in meaningful-pleasant activities (sports, communication, culture/music, and intimacy), we found this to not be the case. Rather, it seems that people with high trait meaningfulness simply judge their everyday activities to be more meaningful than people with low meaning in life, even if both groups find their activities equally pleasant. Alternatively, it might be the case that people with high trait meaningfulness do not just value these same activities as more meaningful, but that they choose more meaningful ways to do them: for example, they could choose an interesting rather than a boring job, or they choose something fascinating and meaningful to read or watch rather than reacting to notifications on social media or zapping on TV. Further research is necessary to clarify this. In summary, these new results provide further empirical evidence for the Hierarchical Meaning Model by Schnell (2009, 2014, 2021): Meaning in life has a top-down effect on our activities and on our perception. Because the assessment of trait meaningfulness chronologically preceded the ESM study, a strong meaning in life can be said to cause (in the sense of Granger causality; Granger, 1969), the experience of daily activities as meaningful.

### Limitations

The results reported here are based on a sample of young, educated, white people. Therefore, they cannot be generalized to other populations. Most participants reported a relatively high trait meaningfulness. A possible explanation for the insignificant results in the Johnson Neyman analysis for trait meaningfulness scores lower than 1.17 might be the fact that only very few people had such low levels of trait meaningfulness. It is striking, however, that the participants experienced a large part of their activities as meaningless. This raises the question of whether this is typical for students, for the specific age group, or other reasons. A replication of the study using a heterogeneous sample would therefore be useful. Another limitation is the assessment of state pleasantness by one item only. Although the reliability of this measure might thus be questionable, the use of single items for narrow and unambiguous constructs is increasingly seen as acceptable (*cf.* Allen et al., 2022). Moreover, several activities like intimacy (*n* = 30), sports (*n* = 35), and reading (*n* = 46) were observed on a low incidence basis. Further replications should thus validate these results.

Finally, the data were collected through self-report questionnaires, and it is not clear how much the subjects were biased by the survey itself. In most cases, people are unaware of the meaningfulness of their lives, and meaning becomes questionable primarily only in times of crisis (Schnell, 2021). By repeatedly asking about the meaningfulness of activities, several times a day and over the course of a week, an artificial situation is created that does not normally occur among people who are not in a crisis of meaning.

### Conclusion

Returning to the initial question of how modern individuals can achieve a good life without the guidance of external authorities, it seems less relevant *what* activities they perform in their everyday life. Instead, it seems more important to know *why* they do them, in order to be able to frame them in a generally present sense of meaning in life. What matters is therefore to develop one’s personal meaning in life. For individuals in modern, highly individualistic societies, condemned to freedom (Sartre and König, 2014), meaning in life is no longer an object to be found in sacred texts, social norms, or traditions, but an attitude to be trained and elaborated. If they then manage to enjoy what they are doing as well, days full of good moments are not only possible, but very likely.

## Data availability statement

The raw data supporting the conclusions of this article will be made available by the authors, without undue reservation.

## Ethics statement

Ethical review and approval was not required for the study on human participants in accordance with the local legislation and institutional requirements. The patients/participants provided their written informed consent to participate in this study.

## Author contributions

CK analyzed the data and wrote the first draft of the manuscript. TS conceptualized the study, collected the data, and revised the manuscript. Both authors contributed to the article and approved the submitted version.

## Conflict of interest

The authors declare that the research was conducted in the absence of any commercial or financial relationships that could be construed as a potential conflict of interest.

## Publisher’s note

All claims expressed in this article are solely those of the authors and do not necessarily represent those of their affiliated organizations, or those of the publisher, the editors and the reviewers. Any product that may be evaluated in this article, or claim that may be made by its manufacturer, is not guaranteed or endorsed by the publisher.

## References

[ref1] Allen M. S. Iliescu D. Greiff S. (2022). Single item measures in psychological science: a call to action [editorial]. Eur. J. Psychol. Assess. 38, 1–5. doi: 10.1027/1015-5759/a000699

[ref2] Antonakis J. Bastardoz N. Rönkkö M. (2021). On ignoring the random effects assumption in multilevel models: review, critique, and recommendations. Organ. Res. Methods 24, 443–483. doi: 10.1177/1094428119877457

[ref001] Bates D. Maechler M. Bolker B. Walker S. Christensen R. H. B. Singmann H. (2018). Package ‘lme4’. Available at: https://cran.r-project.org/web/packages/lme4/lme4.pdf

[ref3] Bechara A. (2005). Decision making, impulse control and loss of willpower to resist drugs: a neurocognitive perspective. Nat. Neurosci. 8, 1458–1463. doi: 10.1038/nn1584, PMID: 16251988

[ref4] Choi J. Catapano R. Choi I. (2017). Taking stock of happiness and meaning in everyday life: an experience sampling approach. Soc. Psychol. Personal. Sci. 8, 641–651. doi: 10.1177/1948550616678455

[ref5] Christensen J. F. (2017). Pleasure junkies all around! Why it matters and why ‘the arts’ might be the answer: a biopsychological perspective. Proc. Biol. Sci. 284:20162837. doi: 10.1098/rspb.2016.2837, PMID: 28469018 PMC5443939

[ref6] Chu S. T.-W. Fung H. H. Chu L. (2020). Is positive affect related to meaning in life differently in younger and older adults? A time sampling study. J. Gerontol. B Psychol. Sci. Soc. Sci. 75, 2086–2094. doi: 10.1093/geronb/gbz086, PMID: 31251360

[ref7] Damásio B. F. Koller S. H. Schnell T. (2013). Sources of meaning and meaning in life questionnaire (SoMe): psychometric properties and sociodemographic findings in a large Brazilian sample. Acta de Investigación Psicológica 3, 1205–1227. doi: 10.1016/S2007-4719(13)70961-X

[ref8] Darandari E. Z. (2004). Robustness of hierarchical linear model parameter estimates under violations of second-level residual homoskedasticity and independence assumptions. Doctoral dissertation. Tallahassee, FL: The Florida State University.

[ref9] Delle Fave A. Massimini F. Bassi M. (2011). Psychological Selection and Optimal Experience Across Cultures: Social Empowerment Through Personal Growth. Vol. 2. Berlin, Germany: Springer Science & Business Media.

[ref10] Ford B. Q. Mauss I. B. Gruber J. (2015). Valuing happiness is associated with bipolar disorder. Emotion 15, 211–222. doi: 10.1037/emo0000048, PMID: 25603134 PMC4380777

[ref11] Ford B. Q. Shallcross A. J. Mauss I. B. Floerke V. A. Gruber J. (2014). Desperately seeking happiness: valuing happiness is associated with symptoms and diagnosis of depression. J. Soc. Clin. Psychol. 33, 890–905. doi: 10.1521/jscp.2014.33.10.890, PMID: 25678736 PMC4321693

[ref12] George D. Mallery P. (2020). IBM SPSS Statistics 26 Step by Step. A Simple Guide and reference. 16th Edn. London, United Kingdom: Routledge.

[ref13] Granger C. W. J. (1969). Investigating causal relations by econometric models and cross-spectral methods. The Econ. Soc. 37, 424–438. doi: 10.2307/1912791

[ref14] Gross P. (1994). Die Multioptionsgesellschaft. Berlin, Germany: Suhrkamp Verlag.

[ref004] Hadley W. (2016). Ggplot2: Elegant graphics for data analysis. Use R!. New York: Springer.

[ref15] Halusic M. King L. A. (2013). “What makes life meaningful: positive mood works in a pinch,” in The Psychology of Meaning. eds. Markman K. D. Proulx T. Lindberg M. J. (Washington, DC: American Psychological Association), 445–464.

[ref16] Hanfstingl B. (2013). Ego and spiritual transcendence: relevance to psychological resilience and the role of age. Evid. Based Complement. Alternat. Med. 2013:949838. doi: 10.1155/2013/949838, PMID: 24223619 PMC3810183

[ref17] Hicks J. A. Trent J. Davis W. E. King L. A. (2012). Positive affect, meaning in life, and future time perspective: an application of socioemotional selectivity theory. Psychol. Aging 27, 181–189. doi: 10.1037/a0023965, PMID: 21707177

[ref18] Huta V. Ryan R. M. (2010). Pursuing pleasure or virtue: the differential and overlapping well-being benefits of hedonic and eudaimonic motives. J. Happiness Stud. 11, 735–762. doi: 10.1007/s10902-009-9171-4

[ref19] Kashdan T. B. Breen W. E. (2007). Materialism and diminished well–being: experiential avoidance as a mediating mechanism. J. Soc. Clin. Psychol. 26, 521–539. doi: 10.1521/jscp.2007.26.5.521

[ref20] King L. A. Hicks J. A. (2021). The science of meaning in life. Annu. Rev. Psychol. 72, 561–584. doi: 10.1146/annurev-psych-072420-122921 32898466

[ref21] King L. A. Hicks J. A. Krull J. L. Del Gaiso A. K. (2006). Positive affect and the experience of meaning in life. J. Pers. Soc. Psychol. 90, 179–196. doi: 10.1037/0022-3514.90.1.179 16448317

[ref22] Klinger E. (1998). “The search for meaning in evolutionary perspective and its clinical implications,” in The Human Quest for Meaning: A Handbook of Psychological Research and Clinical Application. eds. Wong P. T. P. Fry P. S. (New York, NY: Lawrence Erlbaum Associates), 27–50.

[ref23] Long J. A. Long M. J. A. (2019). Package ‘interactions’. Available at: https://interactions.jacob-long.com

[ref25] Martela F. Ryan R. M. Steger M. F. (2018). Meaningfulness as satisfaction of autonomy, competence, relatedness, and beneficence: comparing the four satisfactions and positive affect as predictors of meaning in life. J. Happiness Stud. 19, 1261–1282. doi: 10.1007/s10902-017-9869-7

[ref26] Mauss I. B. Savino N. S. Anderson C. L. Weisbuch M. Tamir M. Laudenslager M. L. (2012). The pursuit of happiness can be lonely. Emotion 12, 908–912. doi: 10.1037/a0025299, PMID: 21910542

[ref27] Mauss I. B. Tamir M. Anderson C. L. Savino N. S. (2011). Can seeking happiness make people unhappy? Paradoxical effects of valuing happiness. Emotion 11, 807–815. doi: 10.1037/a0022010, PMID: 21517168 PMC3160511

[ref28] Miao M. Gan Y. (2020). The promotional role of meaning in life in future-oriented coping: positive affect as a mediator. Int. J. Psychol. 55, 52–59. doi: 10.1002/ijop.12543, PMID: 30362105

[ref29] Newman D. B. Schwarz N. Stone A. A. (2021). Global reports of well-being overestimate aggregated daily states of well-being. J. Posit. Psychol. 16, 407–416. doi: 10.1080/17439760.2020.1725608, PMID: 34025746 PMC8132649

[ref002] Pinheiro J. Bates D. DebRoy S. Sarkar D. Heisterkamp S. van Willigen B. (2017). Package ‘nlme’. Linear and nonlinear mixed effects models, version, 3. Available at: http://cran.rapporter.net/web/packages/nlme/nlme.pdf

[ref003] Revelle W. (2018). psych: Procedures for psychological, psychometric, and personality research. R package version, 1. Available at: http://www2.uaem.mx/r-mirror/web/packages/psych

[ref30] Rolls E. T. (2015). Limbic systems for emotion and for memory, but no single limbic system. Cortex; a journal devoted to the study of the nervous system and behavior 62, 119–157. doi: 10.1016/j.cortex.2013.12.005 24439664

[ref31] Sartre J.-P. König T. (eds.) (2014). “Gesammelte Werke in Einzelausgaben: Philosophische Schriften: Bd. 3,” in Das Sein und das Nichts: Versuch einer phänomenologischen Ontologie. 18th Edn ed (Hamburg, Germany: Rowohlt).

[ref32] Schnell T. (2009). The sources of meaning and meaning in life questionnaire (SoMe): relations to demographics and well-being. J. Posit. Psychol. 4, 483–499. doi: 10.1080/17439760903271074

[ref33] Schnell T. (2010). Existential indifference: another quality of meaning in life. J. Humanist. Psychol. 50, 351–373. doi: 10.1177/0022167809360259

[ref34] Schnell T. (2014). “An empirical approach to existential psychology: meaning in life operationalized,” in Perspectives on Cognitive Psychology. Conceptions of Meaning. ed. Kreitler S. (Hauppauge, NY: Nova Publishers), 173–194.

[ref35] Schnell T. (2021). The Psychology of Meaning in Life. London, United Kingdom: Routledge.

[ref36] Schnell T. (2022). “Suffering as meaningful choice. An existential approach,” in Ta vare. En bok om diakoni, sjelesorg og eksistensiell helse. eds. Austad A. Danbolt L. J. (Oslo: VID), 3–14.

[ref37] Schnell T. Becker P. (2007). Der Fragebogen zu Lebensbedeutungen und Lebenssinn (LeBe). Göttingen, Germany: Hogrefe.

[ref38] Schnell T. Krampe H. (2020). Meaning in life and self-control buffer stress in times of COVID-19: moderating and mediating effects with regard to mental distress. Front. Psychiatry 11:582352. doi: 10.3389/fpsyt.2020.582352, PMID: 33173525 PMC7538834

[ref39] Schnell T. Krampe H. (2022). Meaningfulness protects from and crisis of meaning exacerbates general mental distress longitudinally. BMC Psychiatry 22:285. doi: 10.1186/s12888-022-03921-3, PMID: 35448989 PMC9023037

[ref40] Schueller S. M. Seligman M. E. (2010). Pursuit of pleasure, engagement, and meaning: relationships to subjective and objective measures of well-being. J. Posit. Psychol. 5, 253–263. doi: 10.1080/17439761003794130

[ref41] Skov M. Nadal M. (2018). Art is not special: an assault on the last lines of defense against the naturalization of the human mind. Rev. Neurosci. 29, 699–702. doi: 10.1515/revneuro-2017-0085 29373323

[ref005] Sørensen T. La Cour P. Danbolt L. J. Stifoss-Hanssen H. Lien L. DeMarinis V. (2019). The Sources of Meaning and Meaning in Life Questionnaire in the Norwegian Context: Relations to Mental Health, Quality of Life, and Self-Efficacy. Int. J. Psychol. Religion 29, 32–45. doi: 10.1080/10508619.2018.1547614

[ref42] Stavrova O. Luhmann M. (2016). Social connectedness as a source and consequence of meaning in life. J. Posit. Psychol. 11, 470–479. doi: 10.1080/17439760.2015.1117127

[ref006] Tov W. Lee H. W. (2016). A closer look at the hedonics of everyday meaning and satisfaction. J. Pers. Soc. Psychol. 111, 585–609. doi: 10.1037/pspp0000081 26619303

[ref43] Vötter B. Schnell T. (2019a). Bringing giftedness to bear: Generativity, meaningfulness, and self-control as resources for a happy life among gifted adults. Front. Psychol. 10:1972. doi: 10.3389/fpsyg.2019.01972, PMID: 31572251 PMC6753398

[ref44] Vötter B. Schnell T. (2019b). Cross-lagged analyses between life meaning, self-compassion, and subjective well-being among gifted adults. Mindfulness 10, 1294–1303. doi: 10.1007/s12671-018-1078-x

[ref007] Wickham H. Averick M. Bryan J. Chang W. McGowan L. François R. (2019a). Welcome to the Tidyverse. J. Open Source Softw. 4:1686. doi: 10.21105/joss.01686

[ref008] Wickham H. Francois R. Henry L. Müller K. (2019b). Package ‘dplyr’. A grammar of data manipulation. R Package version 0.8.0.1, 1–88. Available at: https://cran.r-hub.io/web/packages/dplyr/dplyr.pdf

